# Grape Polyphenols’ Effects in Human Cardiovascular Diseases and Diabetes

**DOI:** 10.3390/molecules22010068

**Published:** 2017-01-01

**Authors:** Zuriñe Rasines-Perea, Pierre-Louis Teissedre

**Affiliations:** 1 Université de Bordeaux, ISVV, Institut des Sciences de la Vigne et du Vin, EA 4577 Œnologie, 210 Chemin de Leysotte, Villenave d’Ornon F-33140, France; zrasines@hotmail.es; 2 INRA, Instiut National de la Recherche Agronomique, ISVV, Institut des Sciences de la Vigne et du Vin, USC 1366 Œnologie, 210 Chemin de Leysotte, Villenave d’Ornon F-33140, France

**Keywords:** grape polyphenols, cardiovascular diseases, type 2 diabetes, dietary antioxidant

## Abstract

The consumption of fruits and vegetables, as well as foods enriched in bioactive compounds and nutraceuticals, has increased due to consumers’ interest in the relevance of food composition for human health. Considerable recent interest has focused on bioactive phenolic compounds in grape, as they possess many biological activities, such as antioxidant, cardioprotective, anticancer, anti-inflammation, anti-ageing and antimicrobial properties. Observational studies indicate that the intake of polyphenol-rich foods improves vascular health, thereby significantly reducing the risk of hypertension, and cardiovascular disease (CVD). Other researchers have described the benefits of a grape polyphenol-rich diet for other types of maladies such as diabetes mellitus. This is a comprehensive review on the consumption of polyphenolic grape compounds, concerning their potential benefits for human health in the treatment of cardiovascular diseases and diabetes.

## 1. Introduction

Since ancient times wine has been closely associated with the diet, particularly in Mediterranean countries [1], and for many years, moderate and regular consumption of wine has been associated with health benefits, with no scientific basis. Over the last two decades, several epidemiological and clinical studies around the world have pointed out that the moderate intake of alcoholic beverages produces positive effects on antioxidant capacity, lipid profile and the coagulation system [2], that may be associated with the decreased incidence of cardiovascular disease (CVD) [3,4], overall mortality [5] and other diseases observed in such moderate drinkers.

Several studies have focused their attention on the components of red wine (mainly polyphenols and especially resveratrol) since the so-called “French paradox” was first described [6] in order to explain the relationship observed between wine consumption and the incidence of CVD. Although the chemical constituents of grapes and wine may vary, similar beneficial effects have been observed in different varieties of red wine related to their higher polyphenolic content.

**Figure 1:**
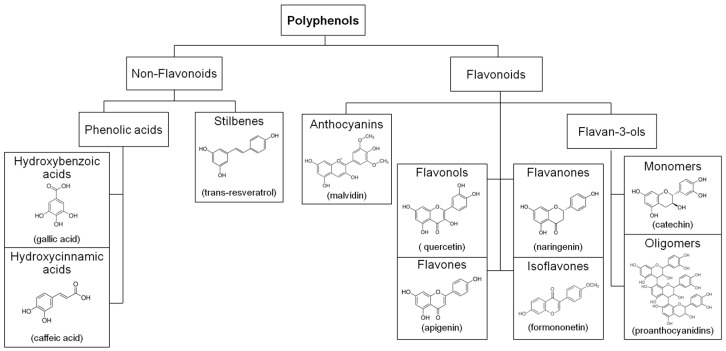
Polyphenol subgroup hierarchy and structure examples.

Polyphenols are the most abundant secondary metabolites present in the plant kingdom. They represent a large and diverse group of molecules including two main families (Figure 1): the flavonoids based on common C_6_-C_3_-C_6_ skeleton and the non-flavonoids.

Dietary intake of polyphenols is highly variable. A study in 1976 estimated the intake at 1 g of glycosylated flavonoids per day in the United States of America (USA) [7]. Some years after, in 1987–1988 a published Dutch study established lower amounts of flavanols and flavones of approximately 23 mg/day [8]. Recently, in 2007, the baseline mean intake of flavonols and flavone was 21.2 mg/day, with quercetin (15.4 mg/day) being the major contributor for United States (US) women [9]. The daily intake of anthocyanins in the USA is estimated to be 12.5 mg/day per person, with delphinidin contributing approximately 21% of the total anthocyanin intake [10]. An accurate estimate of dietary intake of polyphenols is difficult to achieve because of the poor characterization of polyphenols in foods and the great variability of polyphenol content within foods [11].

There is another disease related with CVD and heart failure, which is diabetes mellitus. The major factor in the development of diabetes is obesity. Besides this factor, a number of other risk factors increase the likelihood of developing diabetes. The US Centers for Disease Control and Prevention (CDC) has described the risk factors for developing diabetes, which include physical inactivity, an immediate relative with diabetes, abnormal cholesterol and triglyceride levels, and high blood pressure [12], similar symptoms to develop a CVD.

The objective of this paper is to review the benefits of different grape polyphenols in in vivo and ex vivo treatments related with CVDs and diabetes.

## 2. Cardiovascular Disease

As several reviews indicate [13,14], dietary intake of flavonoids decreases the risk of CVD. Moreover, the established and emerging cardioprotective effects of grapes, which are rich in flavonoids, are described in some reviews [15,16].

### 2.1. Blood Pressure (BP)

Currently, hypertension is related with a high blood pressure value. The major risk factor for the development of CVD [17,18], a disease that ranks the highest among of non-communicable diseases (NCD) [19]. Anti-hypertensive were the most dispensed drugs (698 million prescriptions) in the US during 2012 and 2013 [20]. As a result, preventative strategies and resourceful management of hypertension that can be used by everyone are urgently needed.

This hypertension symptom can be mitigated by dietary practices [21,22]. The risk of stroke mortality can be reduced by 10% with a 2 mm Hg lower usual systolic blood pressure (SBP), as well as the mortality from ischemic heart disease or other vascular causes decreased by 7% in middle-aged persons [23].

Different studies with grapes have included BP measurements [24,25,26,27,28,29,30,31,32,33] that have been conducted with cohorts whose average baseline BP was also in the pre-hypertensive range. The studies of grape seed extracts (GSE) achieved by Sivaprakasapillai et al. [27], in which 27 adults with metabolic syndrome were randomized into three groups (placebo, 300 mg per day of GSE and 150 mg per day of GSE) and those carried out by Clifton [28], a double-blind randomized crossover control trial with a 12 weeks long period on 36 men and women with above-average vascular risk, described a decrease in SBP of −11 mm Hg and −3 mm Hg, respectively. Moreover, Sivaprakasapillai et al. [27] have also observed the same tendency for diastolic blood pressure (DBP), with a decrease from −7 to −11 mm Hg with two different dosages. Studies of nine randomized control trials (RCTs) showed that GSE significantly lowered SBP by −1.54 mm Hg (*p* = 0.02), but no significant effect was observed on DBP [34]. In contrast, a double blind RCT 8-week intervention study carried out by Ras et al. [26], with seventy pre- and stage 1 hypertensive subjects who consumed either 300 mg/day of GSE or a placebo, indicated a change in SBP values in both groups (−5.2 mm Hg and −2.2 mm Hg, *p* = 0.01, for GSE and placebo groups, respectively). The DBP was also changed by −2.5 mm Hg (*p* = 0.01) in the GSE group and by −1.1 (*p* = 0.01) mm Hg in the placebo group. Another study by Draijer et al. [35] has demonstrated that consumption of a polyphenol-rich grape-wine extract containing 800 mg of polyphenols lowered SBP by −3 mm Hg and DBP by −2 mm Hg in 60 untreated mildly hypertensive subjects and also confirmed that 24-h ambulatory BPs were significantly lower in the grape-wine extract intervention (135.9 ± 1.3 mmHg), compared to placebo (138.9 ± 1.3 mmHg).

Contrarily, three studies found no changes in BP values during their experiments [29,30,31], although the studies were performed on healthy people or individuals with pre- and stage 1 hypertension.

A RCT study published in 2015 by Vaisman et al. [36] which investigated the effect of daily dietary consumption of red grape cell powder (RGC) on blood pressure in 50 subjects with prehypertension and mild hypertension who consumed 200, 400 mg RGC or placebo daily for 12 weeks, described a significant decrease of DBP in the 200 mg RGC group compared to the placebo group (*p* = 0.032). A more recent study carried out by Biesinger et al. [37] showed a decrease in DBP of 4.4 mmHg and unchanged SBP when 18 hypertensive people with metabolic syndrome were treated during 28 days with a polyphenol mix comprised of GSE (330 mg), green tea (100 mg), resveratrol (60 mg) and a combination of bilberry, quercetin and ginkgo biloba (see Table 1 for design description information and significant results).

Although RCTs need to be conducted to determine if grapes have an effect on BP in pre- and stage 1 hypertensive patients, they have shown promise in reducing BP, mostly in individuals with higher usual BP.

### 2.2. Blood Lipids

Although high cholesterol can be treated with dietary measures or pharmaceutical interventions before vascular injuries and CVD develops, it is a factor that is not usually given appropriate importance. Having high concentrations of low-density lipoprotein cholesterol (LDL-C) and reduced values of high-density lipoprotein cholesterol (HDL-C) are important factors for CVD [38,39].

Red wine affects cholesterol by several mechanisms whereby polyphenols participate in hepatic cholesterol and lipoprotein metabolism. This mechanism works by reducing cholesterol absorption and decreasing the delivery of cholesterol to the liver, which in turn reduces plasma cholesterol. Additionally, polyphenols affect apolipoproteins (apo) A and B, which are emerging as risk factors for CVD [40], modify Very Low Density Lipoproteins (VLDL) particles and reduce plasma triglyceride (TG) levels due to possible increased lipoprotein lipase (LPL) activity, which leads to decreased LDL in the circulation [41].

Evidences provided by Castilla et al. [42,43], Khadem-Ansari et al. [44] and Albers et al. [45] suggest that grape juice can have an effect on blood lipids. Studies conducted by Castilla et al. using 100 mL/day of concentrated Bobal grape juice for 14 days shown a decrease in total cholesterol (TC), LDL-C and apo B-100 (*p* < 0.001) in 38 [42] and 32 [43] healthy and hemodialysis patients, whereas the HPL-C and apo A-1 values increased (*p* < 0.001 [42] and *p* < 0.01 [43]).

Two other studies with grape juice [44,45], both resulted in increased HLD-C (*p <* 0.0001 [44] and *p* = 0.02 [45], respectively), and also an augmentation of apo B (*p* < 0.002) in the Khadem-Ansari et al. [44] study. Two additional studies with Concord grape juice found an increase in TG (*p* < 0.001 [46] and *p* < 0.05 [47]) and no change in TC, HDL-C or LDL-C.

Other articles have described different results depending on grape extract administration and the patients’ disease. In this context, no changes in LDL-C or TG were found in 24 overweight pre-hypertensive and/or pre-diabetic individuals, although an increase in HDL-C and a decrease in TC were achieved (*p* = 0.001 and *p* = 0.037, respectively), when the individuals were supplemented with 350 mg/day of whole grape extract for 6 weeks [48], whereas in a study with 52 mild hyperlipidemic individuals supplemented with 200 mg/day of GSE, no change was observed in HDL-C or TG. Indeed, decreases in TP and LDL-c were found (*p* = 0.015 and *p* = 0.014, respectively) [49]. Another long-term human supplementation study with 2 × 300 mg/day of MegaNatural (R) Gold during 3 weeks found that TC, LDL-C and HDL-C concentrations were significantly decreased in eight hypercholesterolemic subjects (TC and LDL-C, *p* < 0.01; HDL-C, *p* < 0.05), while no effect was found in nine normal individuals [50] (see Table 2 for design description information and significant results).

Concerning resveratrol (RES) experiments, a randomized, placebo-controlled crossover study was conducted by van der Made et al. [51] in 45 overweight and slightly obese men and women. Subjects received 150 mg/day of resveratrol or placebo capsules for 4 weeks. No difference between resveratrol and placebo was found in HDL-C or LDL-C. One year before, Magyar et al. [52] observed an improvement in endothelial function measured by flow-mediated vasodilation (FMD) (*p* < 0.05) LDL level (*p* < 0.05), studying the effects of RES in a double-blind, placebo-controlled trial with 40 post-infarction patients who received 10 mg of RES per day for 3 months.

### 2.3. LDL Oxidation and Oxidative Stress

Polyphenols, especially those found in grape products such as red wine and grape juice, inhibit LDL oxidation and thus attenuate the development of atherosclerosis [45,53,54,55,56,57,58]. Studies with hemodialysis patients associated with a consumption of 100 mL/d concentrated Bobal grape juice for 14 days [42,43] observed decreases of 35% and 65% in oxidized LDL (ox-LDL), respectively. Studies conducted with Concord grape juice found discrepant results; the first one in 1999 found a 35% increase in LDL lag time (*p* = 0.015) with stable atherosclerotic coronary artery disease patients after consuming 7.7 mL/kg per day during 14 days [46]. On the other hand, the study by O’Byrne et al. in 2002 observed a 10% increase in LDL lag time (*p* < 0.001) and a 9% decrease in LDL oxidation rate (*p* < 0.01) in healthy individuals after consuming 10 mL/kg per day during 14 days [47]. A recent study with *Vitis Labrusca* L. grape juice in which 24 healthy subjects participated in a randomized, controlled, crossover study based on three different regimes [59]: (1) acute consumption of 400 mL of conventional red grape juice (Bordo/Isabel); (2) 400 mL of organic red grape juice (Bordo); (3) 400 mL of water (control); demonstrated that the ingestion of *Vitis Labrusca* L. grape juices promoted a significant decrease in thiobarbituric acid reactive substances (TBARS) levels when compared to the control intervention (*p* < 0.05).

Some studies have been conducted with grape seed extract to analyze LDL oxidation. The first one, published in 2003, was carried out by Vigna et al. [60] with 24 healthy male heavy smokers during 4 weeks of treatment with two capsules daily of 75 mg of a grape procyanidin extract. Among oxidative index results achieved, TBARS concentration was significantly reduced in subjects taking the standardized formulation of a polyphenolic extract of grapes versus placebo treatment (−14.7% ± 21.1% vs. +5.0% ± 18.1%, *p* < 0.01). Similarly, the lag phase of LDL oxidation was prolonged with respect to baseline (+15.4% ± 24.4% after procyanidins and +0.1% ± 16.0% after placebo, *p* < 0.05). On the other hand, a study carried out by Ward et al. in 2005 [31], found no change in ox-LDL levels after a randomized, double-blind, placebo controlled, factorial trial in which 18 hypertensive individuals were treated with 1000 mg/day grape seed extract during 6 weeks.

The research published in 2007 by Sano et al. [29] examined the effect on healthy individuals of 200 or 400 mg/day administration of proanthocyanidin equivalent for 12 weeks. Both dosages decreased the concentration of ox-LDL, measured as plasma maloaldehyde (MDA) LDL, by 12%–14% (*p* < 0.05). The last study published in 2013 by Razavi et al. [49] found a decrease in ox-LDL (*p* = 0.008) after 8 weeks treatment with 200 mg/day of red grape seed extracts in individuals with mild hyperlipidemia.

In 2012 a study was carried out by Tomé-Carneiro et al. [61], in which 75 patients undergoing primary prevention of cardiovascular disease participated in a tripled-blinded, randomized, placebo-controlled trial with resveratrol-enriched grape extract (8 mg of resveratrol) during 6 months. After the treatment, decreases in LDL-C (*p* = 0.04), Apo-B (*p* = 0.014) and ox-LDL (*p* = 0.001), were observed, reducing atherogenic markers. The presence of resveratrol in the GE was necessary to achieve these effects.

Oxidative stress reflects an imbalance between the systemic manifestation of reactive oxygen species and a biological system’s ability to readily detoxify the reactive intermediates or to repair the resulting damage. The generation of free radicals as superoxide (O_2_^.−^), hydrogen peroxide (H_2_O_2_), hydroxyl radical (OH^-^), peroxynitrite (ONO_2_^−^), and nitric oxide (NO) is necessary, as they have roles in growth, repair, and immune functions that are essential for human cells. In contrast, these molecules also have the ability to oxidize signalling molecules, DNA, macromolecules, and cell structures such as lipid membranes of healthy cells, all of which are to the detriment of these cells.

Long-term oxidative stress states have been linked to various diseases as diabetes, chronic obstructive pulmonary disease (COPD), cardiovascular disease, cancer and asthma. Several human studies have documented decreases in markers of oxidative stress, as superoxide and F2-isoprostanes, following supplementation with grapes. A reduction in superoxide anion production, which is involved in atherogenesis through the formation of ox-LDL [62], has been found relative to both neutrophils and platelets after grape supplementation. In 2001, Freedman et al. [63] found significant decreases in levels of platelet-dependent superoxide (from 29.5 ± 5.0 to 19.2 ± 3.1, *p* < 0.05) among 20 healthy subjects who consumed 7 mL/kg per day of purple grape juice for 14 days. Similarly, in 2004 Albers et al. [45] found the same tendency for platelet-dependent superoxide (50 au after placebo vs. 34.5 au after Concord grape juice, *p* = 0.02) after 14 days of supplementation with 7 mL/kg per day of Concord grape juice in a study with 20 subjects with previously diagnosed coronary disease. In 2008, Castilla et al. [43] described a decrease in NADPH oxidase-dependent production of superoxide by neutrophils (*p* < 0.01) in stable hemodialysis patients after 14 days of 100 mL of concentrated Bobal grape juice.

When it comes to F2-isoprostanes as an indicator of oxidative stress [64], controversial results were found. In 2001, Caccetta et al. [65] described decreases in F-isoprostane value (*p* < 0.05) in plasma and in urine with dealcoholized red wine in a RCT with 18 smokers. One year later, O’Byrne et al. [47] published a study in with healthy young adults consumed 10 mL/kg per day of grape juice for 2 weeks, and no change in urinary F2-isoprostanes was found. In 2005, Zern et al. [66] reported a decrease in urinary F2-isoprostanes (*p* < 0.05) after 4 weeks of treatment with 36 g of lyophilized grape powder (~200 g fresh) in pre- and post-menopausal women. Ward et al. [31] found no change in plasma or urinary F2 isoprostanes after hypertensive individuals were supplemented with 1000 g/day of grape seed extract for 6 weeks. In 2013, Hokayem et al. [67] found an increase in systemic oxidative stress expressed as higher values of urinary F2-isoprostanes after 8 weeks of 2 g/day grape polyphenol supplementation in 38 healthy overweight/obese first degree relatives of type 2 diabetic patients in a randomized double-blind controlled trial.

There are two studies conducted to examine DNA damage as a biomarker of oxidative stress. The first one, with a supplementation of 5.5 mL/kg per day of Concord grape juice in hypertensive men [68], observed decreases in lymphocyte DNA damage (*p* < 0.01) after 8 weeks of treatment. Recently, Corredor et al. [69] observed a significant decrease when chronic kidney disease patients in dialysis were supplemented with 100 mL of unfermented grape juice in each dialysis session after 6 months of treatment. All supplement trial information is collected in Table 3.

### 2.4. Platelet Activation

Platelets are involved in atherosclerotic coronary artery disease (CAD) development, and inhibition of platelet aggregation (PA) is an accepted mechanism in cardioprotection. There are not so many in vivo or ex vivo studies with grapes, although they suggest that they have a beneficial effect on platelet aggregation. The first ex vivo study was published in 1996 [70]. This study was performed on 24 healthy males aged 26–45 years who consumed red wine, commercial white wine grape juice and the same juice enriched with *trans*-resveratrol for periods of 4 weeks, in which an increase of PA response to thrombin (*p* < 0.02 and *p* < 0.001, respectively) it was observed, but only white wine increased the PA response to adenosine diphosphate (ADP, *p* < 0.05). The commercial juice lowered the AP response to thrombin (*p* < 0.001) whereas the resveratrol-enriched juice caused a dramatic increase (*p* < 0.001). In 1998, Watkins and Bierenbaum [71] observed that 180 mL/day of white grape juice decreased PA in response to both collagen and thrombin agonist (*p* < 0.02) after 4 weeks of consumption in hypercholesterolemic individuals, whereas red grape juice did not. In 2000, Keevil et al. [72] published a study that sought to evaluate whether commercial grape, orange and grapefruit juices, taken daily, reduced ex vivo platelet activity, in a randomized cross-over design with ten healthy human subjects. They showed that drinking purple grape juice for one week reduced the whole blood PA response to 1 mg/L of collagen by 77% (from 17.9 ± 2.3 to 4.0 ± 6.8 ohms, *p* = 0.0002), whereas orange juice and grapefruit juice had no effect on platelet aggregation. One year later, Freedman et al. [63] described a decrease in PA to ADP (*p* < 0.01) after 14 days consuming 7 mL/kg of purple grape juice. The differences in the results can be produced by the juice type and quantity consumed, and also the antagonist choice or use of platelet-rich plasma aggregation.

### 2.5. Atrial Fibrillation

Artrial fibrillation (AF) is the most common type of cardiac electrical rhythm disturbance, with a prelevance that increases with age [73]. AF is the primary cause of 15% of strokes [74,75] and can also cause adverse remodeling of the heart leading to heart failure (HF) [74,76,77,78]. Both of them, AF and HF, were called the “two new epidemics of cardiovascular disease” [79], with common risk factors as hypertension, valvular heart disease, coronary artery disease and diabetes [80]. Once stablished, AF can promote HF owing to rapid ventricular rate, leading to tachycardia-induced cardiomyopathy and associated myocardial maladaptive remodeling [76,81,82,83]. These alterations are partly adaptive in nature, but they can lead to further deterioration of cardiac function resulting in HF when maintained for longer periods.

Improved knowledge of the mechanisms underlying AF represents a key opportunity to develop novel atrial-specific therapies for his treatment. Several promising therapeutic targets have been revealed for potential drug development, as the Kv1.5 (I_Kur_), a potassium channel expressed in the atria, but not the ventricles; late sodium current (late I_Na_); overactive I_KACh_ channels [84,85,86]; inflammation/oxidative stress [87]; and activation of the nuclear factor of activated T cells (NFAT) [88]. Targets that have been implicated in the development of AF [89].

Currently, there is only one study that initiated a drug development program using resveratrol as the active parent compound with potential results in targeting several key pathways involved in the development and maintenance of AF [90].

## 3. Diabetes Mellitus

Diabetes mellitus is one of the most significant public health problems in the world. Including undiagnosed cases of diabetes, this number is expected to reach 592 million by 2030 (an increase of 55% of the total population) [91,92]. The WHO has reported that there were about 422 million people worldwide had diabetes in 2014 [93].

Preventing the development of diabetes is actually more cost-effective than treating its manifestation and complications [94]. Recently, some evidences were found suggesting that dietary polyphenols and polyphenol-rich foods are beneficial for preventing and managing diabetes [23,24]. Several excellent reviews have illustrated that natural polyphenols are potential multifunctional agents to reduce the risk of diabetes and diabetic complications [95,96,97,98,99,100].

The carbohydrates from food or meals are the main sources leading to hyperglycemia and hyperinsulinemia in diabetics. The potential of dietary polyphenols for controlling hyperglycemia is a very interesting aspect [101]. Polyphenols’ dietary effects can be summarized as: protection of pancreatic-cells against glucose toxicity, anti-inflammatory and antioxidant effects, inhibition of starch digestion by inhibition of digestion enzymes, improvement of insulin resistance, and inhibition of advanced glycation end products (AGEs) formation [98,102,103]. Most studies to test the health benefits of grape polyphenols for diabetes have been performed using animal models [104,105,106,107,108]. Furthermore, the studies performed to evaluate the effects of human grape polyphenols intake are related only with type 2 diabetes (T2D) [99,109,110,111,112,113,114]. In 2011, Guilford et al. reviewed the relationship between wine and health and revealed that moderate and regular red wine consumption is linked with a 30% risk reduction for T2D [111]. In 2012, an investigation carried out in USA over 20 years reported a relation between the consumption of flavonoids and diabetes. It illustrated that higher intake of anthocyanins was obviously linked to a lower risk for development of type 2 diabetes [112]. In 2013, a meta-analysis on the association between the consumption of flavonoids and the risk of type 2 diabetes was performed [113], suggesting that the intake of total flavonoids is associated with a lower risk for diabetes. Moreover, a van Dam RM et al. study [114] suggested that intake of anthocyanidins and flavan-3-ols may reduce the risk of type 2 diabetes (see Table 4 for design description information and significant results).

Absorption of polyphenols can be affected by dosage, size of phenolic compound, prior diet, food matrix, gender and differences in the gut microbial populations [115]. Indeed, only 5%–10% of the total intake of dietary polyphenols are directly absorbed through the stomach and the small intestine [116]. An increased level of fecal Bifidobacteria has been associated with improved glucose tolerance and diminished inflammatory markers such as the interleukins IL-6, IL-1α and IL-1β, tumor necrosis factor α and monocyte chemoattractant protein-1 [111,117,118]. A study in which a proanthocyanidin-rich extract from grape seeds was administered to nine healthy adults for two weeks increased *Bifidobacterium* but no control material was provided to volunteers [119]. Other study published in the same year by Queipo-Ortuno et al. [120] demonstrated that daily consumption of red wine polyphenols for four weeks increased *Bifidobacterium* compared with baseline, but there was no control for comparison. In addition the *Bifidobacterium* growth was related with a decrease in cholesterol (*p* = 0.012, *R*^2^ = 0.583).

As described before, inflammation and oxidative stress are common in both diseases. In 2009, a double-blinded, randomized, crossover trial of 32 subjects with T2D showed the positive effects of grape seed extract intake of 600 mg/day for four weeks on high sensitivity C reactive protein (hsCRP, *p* = 0.0006), which is an inflammation marker [121]. The study carried out by Hokayem [64] showed protective effects of grape polyphenols on fructose-induced oxidative stress and insulin resistance in first-degree relatives of patients with T2D. After six-day fructose loading, the placebo group showed a 20% decrease in hepatic insulin sensitivity (11.9 ± 1.5 vs. 9.6 ± 0.9; *p* < 0.05) associated with an 11% decrease in glucose infusion rate (*p* < 0.05). However, the grape polyphenol group did not show these deleterious effects of fructose. Moreover, grape polyphenol supplementation showed protective effects against fructose-induced oxidative stress markers of urinary F2-isoprostanes, muscle TBARS [67]. In a RCT carried out by Urquiaga et al. [122] showed a significant reduction (*p* < 0.05) in postprandial insulin and fasting glucose levels compared with the baseline in 38 individuals with at least one component of metabolic syndrome.

Banini et al. showed that continued consumption of dealcoholized muscadine grape wine altered blood insulin levels in type 2 diabetic subjects [123]. Subjects receiving the dealcoholized wine had reduced fasting blood insulin levels and the fasting blood glucose:insulin ratio increased from 8.5 to 13.1 during the 28-day intervention. A low glucose:insulin ratio of <7 is considered predictive of insulin resistance. This study points toward the possibility that consumption of grapes or grape preparations may be beneficial to individuals with aberrant insulin responses to glucose.

Another study was carried out in 2013 by Chiva-Blanch in which 67 men at high cardiovascular risk were randomized in a crossover trial. After a run-in period, all received either red wine (30 g alcohol/day), the equivalent amount of dealcoholized red wine, and gin (30 g alcohol/day) for 4 week periods. Fasting glucose concentrations did not change in any intervention, while the mean adjusted insulin values decreased significantly after the red wine and dealcoholized red wine interventions compared to both baseline (21% and 20%, respectively) and gin groups (15% and 13%, respectively), leading to the conclusion that red wine rich in polyphenols with or without alcohol significantly improves insulin sensitivity, compared with other alcoholic beverages [124].

Concerning RES effects in T2D patients, a study published by Bhatt et al. [125] revealed that supplementation of resveratrol for 3 months significantly improved the mean hemoglobin A_1c_ (*p* < 0.05), SBP (*p* < 0.05), TC (*p* < 0.05), and total protein (*p* < 0.05) levels in a randomized controlled trial with 62 T2D patients who received 250 mg/day of RES for a period of 3 months. In conclusion RES supplementation provides data about the possible clinical effects on the glycemic control and cardiovascular risk factors in patients with T2D.

## 4. Conclusions

The results presented in this review supports the benefits of grape and wine polyphenols in CVD, namely in the areas of inhibition of platelet aggregation, decreased LDL oxidation, and reduction of oxidative stress. Moreover, grapes can also have a favorable effect on blood lipids, decrease inflammation and potentially reduce BP.

There is a paucity of information regarding the health benefits of grapes and grape constituents for management, treatment, or prevention of T2D. Most studies have showed that dietary polyphenols were associated with a lower risk of T2D, but this association was not entirely consistent.

Contradictory results were also shown depending on the intake quantity, type of product (red or white wine, grape juice, grape seed extract, individual component such as resveratrol), treatment period, and number and health condition of individuals in different trials.

**Figure 2:**
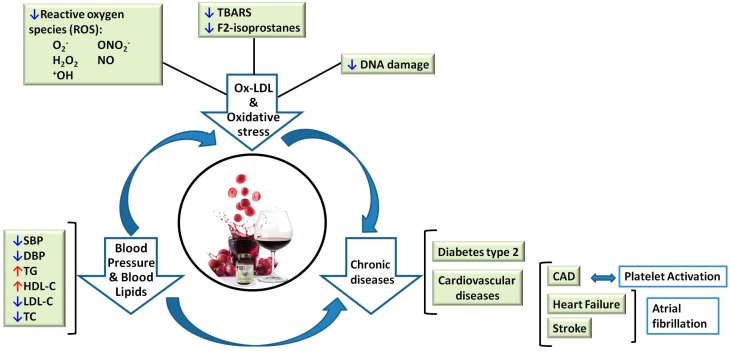
Grape polyphenols moderate consumption effects related with a final chronic disease as Cardiovascular disease or diabetes.

As T2D is a high risk for developing cardiovascular complications and some areas between the two diseases are related, such as plasma triglycerides, cholesterol levels, and inflammatory molecules, they will also be important parameters to examine in future human studies. The relationship between the different points described in this review is shown in Figure 2. Nevertheless, larger and better designed studies are required before any recommendations of intake quantity, period, and type of grape product can be made.

## Figures and Tables

**Table 1:** Human trial design description of grape polyphenols impact in blood pressure.

Study Ref	Subject Description	Trial Type	*n*	Product	Intake (per Day)	Duration	Condition at Testing	Significant Results
Ras et al. 2013 [26]	Healthy	Parallel R PC	70	Grape seed extract	300 mg	8 wk	BP (days 5, 7, 61 and 63) Urine 24 h (days 7 and 63) Blood samples (0 and 2 h after breakfast)	↓ SBP (*p* = 0.01) ↓ DBP (*p* = 0.01)
Sivaprakasapillai et al. 2009 [27]	Metabolic syndrome	Crossover R PC	27 (11 H, 16 F)	Grape seed extract	150 mg 300 mg	4 wk	Fasted	↓ SBP (*p* = 0.05) ↓ DBP (*p* = 0.05) No change in LDL and HDL choresterol ↓ Ox-LDL levels
Clifton 2004 [28]	Above average vascular risk 58 yr	Crossover R PC DB	36 (24 M, 12 F)	Grape seed extract	2 g	4 wk	Fasted	↓ SBP (*p* = 0.05) ↑ FMD (*p* < 0.05)
Sano et al. 2007 [29]	Healthy ≥30 yr and <70 yr	Crossover R PC DB	61 (29 M, 32 F)	Grape seed extract	200 mg 400 mg	12 wk	Fasted at 0, 6 and 12 wk	No significant change in BP, LDL-C, HPL-C ↓ LDL and ox-LDL (12%–14%, *p* < 0.05)
van Mierlo et al. 2010 [30]	Healthy 31.4 ± 9.0 yr	Crossover R PC DB	35 (35 M, 0 F)	Grape seed extract	800 mg	3 periods of 2 weeks	Post-prandial	No change in BP or FMD
Ward et al. 2005 [31]	Hypertensive 61.3 ± 6.3 yr	Parallel PC DB	69	Grape seed extract	1000 mg	6 wk	Fasted	No change in BP or FMD No change in ox-LDL No change in F2-isoprostanes
Vaisman et al. 2015 [36]	Pre- and mild-hypertension ≥35 yr and <70 yr	Parallel R PC DB	50 (35 M, 15 F)	Red grape cell powder	200 mg 400 mg	12 wk	Fasted	↓ DBP (*p* = 0.032) in 200 mg group
Biesinger et al. 2016 [37]	Metabolic syndrome	Crossover R PC DB	18	Combination of extract from seed and skin	330 mg	28 days	Fasted	↓ DBP (*p* = 0.024) No change in SBP

Abbreviations: DB, double blind; F, female; FMD, flow-mediated dilatation measured in brachial artery; M, male; PC, placebo controlled; R, randomized; yr, year; wk, week.

**Table 2:** Human trial design description of grape polyphenols impact in blood lipids.

Study Ref	Subject Description	Trial Type	*n*	Product	Intake (per Day)	Duration	Condition at Testing	Significant Results
Castilla et al. 2006 [42]	Healthy and hemodialysis patients	Crossover R PC	38 (19 H, 19 F)	Red grape juice	100 mL	14 days	Fasted at time 0, 7 and 14	↓ TC, ↓ LDL-C, ↓ apo B-100 (*p* < 0.001); ↑ HPL-C and ↑ apo A-1 (*p* < 0.001) ↓ ox-LDL (35%)
Castilla et al. 2008 [43]	Hemodialysis patients	Crossover R PC	32	Red frape juice	100 mL	14 days	Fasted at time 0, 7 and 14	↓ TC, ↓ LDL-C, ↓ apo B-100 (*p* < 0.001); ↑ HPL-C and ↑ apo A-1 (*p* < 0.01) ↓ ox-LDL (65%) and NADPH (*p* < 0.01)
Khadem-Ansari et al. 2010 [44]	Healthy and non-smokers	Single arm intervention	26 (26 M, 0 F)	Red grape juice	300 mL	1 month	Fasted 12 h before study entry and at the end of study	↑ HLD-C (*p <* 0.0001) and ↑ apo B (*p <* 0.002)
Albers et al. 2004 [45]	CAD patients	Crossover R PC DB	40	Purple grape juice	7 mL/kg	14 days	Fasted	↑ TG *(p <* 0.05) and no change in TC, HDL-C or LDL-C ↓ platelet (*p* = 0.02)
Stein et al. 1999 [45]	CAD 62.5 ± 12.7 yr	Single arm intervention	15 (12 M, 3 F)	Concord grape juice	7.7 ± 1.2 mL/kg	14 days	Fasted (day 0); day 14, 4 mL/kg treatment consumed prior to test; time not given	↑ TG (*p* < 0.001) and no change in TC, HDL-C or LDL-C ↑ LDL (35%)
O’Byrne et al. 2002 [47]	Healthy	Parallel R	36	Concord grape juice	10 mL/kg	2 wk	Fasted	↑ TG (*p* < 0.05) and no change in TC, HDL-C or LDL-C ↑ LDL (10%) and ox-LDL (9%) No change in F2-isoprostanes
Evans et al. 2014 [48]	Pre-hypertensive/pre-diabetic	Crossover R PC DB	24	Grape extract	350 mg	6 wk	Fasted	↑ HDL-C (*p =* 0.001), ↓ TC (*p* = 0.037) and no change in LDL-C or TG.
Razavi et al. 2013 [49]	Mild hyperlipidemic	Crossover R PC DB	52	Grape seed extract	200 mg	8 wk	Fasted	↓ TC (*p* = 0.015), ↓ LDL-C (*p* = 0.014) ↓ ox-LDL (*P* = 0.008))
Vinson et al. 2001 [50]	Healthy and hypercholesterolemic	Parallel R	17	Grape seed extract	600 mg	3 wk	Fasted	↓ TC and ↓ LDL-C, *p* < 0.01; ↓ HDL-C, *p* < 0.05

Abbreviations: C, controlled; CAD, coronary artery disease; DB, double blind; F, female; M, male; PC, placebo controlled; R, randomized; yr, year; wk, week.

**Table 3:** Human trial design description of grape polyphenols impact in LDL oxidation and oxidative stress.

Study Ref	Subject Description	Trial Type	*n*	Product	Intake (per Day)	Duration	Condition at Testing	Significant Results
Toaldo et al. 2015 [57]	Healthy	Crossover R C	24 (5 M, 19 F)	Grape juice	400 mL	2 wk	Fasted	↓ TBARS (*p* < 0.05)
Vigna et al. 2003 [58]	Heavy smokers	Crossover R PC DB	24 (24 M, 0 F)	Grape procyanidin extract	75 mg	4 wk	Fasted	No change in TC, TG, HDL-C and LDL-C ↓ TBARS (*p* < 0.01) and ↑ ox-LDL (*p* < 0.05)
Freedman et al. 2001 [60]	Healthy 30.6 ± 1.8 yr	Single arm intervention	20 (12 M, 8 F)	Purple grape juice	7 mL/kg	14 days	Fasted	↑ Platelet-NO (*P<*0.007) and ↓ Platelet-O2- (*p* < 0.05)
Caccetta et al. 2000 [62]	Smokers	Crossover R	18 (18 M, 0 F)	Red wine, white wine and dealcoholized red wine	375 mL	2 wk	Fasted before and after each bevarage	↓ F2-isoprostanes (*p* < 0.05) with dealcoholized red wine
Zern et al. 2005 [63]	Pre- and post-menopausal women	Crossover R PC	44 (24 F pre-, 20 F post-)	Grape powder	36 g	4 wk	Fasted blood and non fasted urine	↓ TG (*p* < 0.01); ↓ LDL-C, apo-b and apo-e (*p* < 0.05, ↓ F2-isoprastones (*p* < 0.05). No change in ox-LDL
Hokayem et al. 2013 [64]	Healthy overweight/obese T2D	Crossover R PC DB	38 (18 M, 20 F)	Grape polyphenols	2 g	8 wk	Fasted	↓ F2-isopreastones and TBARS (*p* < 0.05) ↓ hepatic insulin (*p* < 0.05) and glucose infusion rate (*p* < 0.05)
Park et al. 2009 [65]	Hypertensive men	Crossover R PC DB	40	Grape juice	5.5 mL/kg	8 wk	Fasted	↓ DNA damage (*p* < 0.01) and ↓ DBP and SBP (*p* < 0.05)
Corredor et al. 2016 [66]	Chronic kidney disease	Crossover R C	39 (21 M, 15 F)	Unfermented grape juice	100 mL	6 months	Blood samples before HD	↓ ox-DNA damage and LDL and C

Abbreviations: C, controlled; DB, double blind; F, female; HD, dialysis; M, male; PC, placebo controlled; R, randomized; yr, year; wk, week.

**Table 4:** Human trial design description of grape polyphenols impact in diabetes.

Study Ref	Subject Description	Trial Type	*n*	Product	Intake (per Day)	Duration	Condition at Testing	Significant Results
Yamakoshi et al. 2001 [116]	Healthy and elderly inpatients	Crossover R	33	Grape seed extract	0.5 g	2 wk	Freshly voided fecal by direct defecation	↑ *Bifidobacterium*
Queipo-Ortuno et al. 2012 [117]	Healthy	Crossover R C	10 (10 M, 0 F)	Red wine and dealcoholized red wine	272 mL	20 days	Fecal samples before and after treatment. Fasted blood and 24-h urine	↑ *Bifidobacterium*
Kar et al. 2009 [118]	T2D subjects 61.8 ± 6.36 yr	Crossover R PC DB	32 (16 M, 16 F)	Grape seed extract	600 mg	4 wk	Fasted	↓ Fructosamine (*p* = 0.0004) ↓ TC (*p* = 0.05) TG and HDL-C stables
Urquiaga et al. 2015 [119]	Metabolic syndrome	Crossover R C	38	Red wine grape pomace	20 g	16 wk	Fasted	↓ Postprandial insulin (*p* < 0.05) ↓ Protein damage
Banini et al. 2006 [120]	T2D subjects	Crossover R C	29	Grape juice, wine and dealcoholized wine	150 mL	28 days	Fasted	↓ blood glucose, insulin and glycated hemoglobin
Chiva-Blanch et al. 2013 [121]	High CVD risk 60 ± 8 yr	Crossover R C	67 (67 M, 0 F)	Red wine and dealcoholized red wine	272 mL	4 wk	Fasted and 24-h urine	↓ insulin value ↓ LDL-C and ↑HLD-C ↓ Apo-B

Abbreviations: C, controlled; CVD, cardio vascular disease; DB, double blind; F, female; M, male; PC, placebo controlled; R, randomized; yr, year; wk, week.
